# Interface Engineering Using Multiple La-Doped HfO_2_ Epitaxial Subnanolayers To Improve the Ferroelectric Properties of Hf_0.5_Zr_0.5_O_2_ Films

**DOI:** 10.1021/acsaelm.5c02016

**Published:** 2025-12-26

**Authors:** Mehrdad Ghiasabadi Farahani, Tingfeng Song, César Magén, Jingye Zou, Florencio Sánchez, Ignasi Fina

**Affiliations:** 1 54449 Institut de Ciència de Materials de Barcelona (ICMAB-CSIC), Campus UAB, Bellaterra 08193, Spain; 2 Department of Applied Physics, The Hong Kong Polytechnic University, Hong Kong 999077, China; 3 Joint Research Center of Microelectronics, The Hong Kong Polytechnic University, Hong Kong 999077, China; 4 Instituto de Nanociencia y Materiales de Aragón (INMA), CSIC-Universidad de Zaragoza, 50009 Zaragoza, Spain; 5 Departamento de Física de la Materia Condensada, 16765 Universidad de Zaragoza, 50018 Zaragoza, Spain

**Keywords:** Ferroelectric, Hafnium oxide, Epitaxy, Multilayers, Nanolaminate, Hf_0.5_Zr_0.5_O_2_

## Abstract

The fabrication of ferroelectric multilayer systems based on hafnia represents a promising approach for achieving high-performance ferroelectric devices. Electrical cycling instability is, in this regard, a key barrier to commercialization. Here, we report on the incorporation of La:HfO_2_ subnanolayers into an epitaxial Hf_0.5_Zr_0.5_O_2_ film, forming multilayer heterostructures. Ferroelectric properties of multilayers are compared with single-layer structures. We observe that wake-up and fatigue are not present up to 10^5^ cycles in the multilayers. The improved stability is enabled by the ≈25% reduction of coercive field together with the lower leakage resulting from the columnar microstructure throughout the entire thickness without phase discontinuity at interfaces and negligible presence of structural defects. This improvement on endurance response is obtained while the polarization is maintained in comparison with single Hf_0.5_Zr_0.5_O_2_ films; there is negligible loss of the polarization throught time and there is a fast response time lower than 100 ns, limited by the measurement circuit. In addition, dielectric permittivity and large resistive switching up to 10^8^%, not related to the ferroelectric response, are also observed. These findings underscore multilayer architecture as an interesting approach to improve properties while also showing that careful selection of interlayer composition is critical to improve device performance.

## Graphical abstract

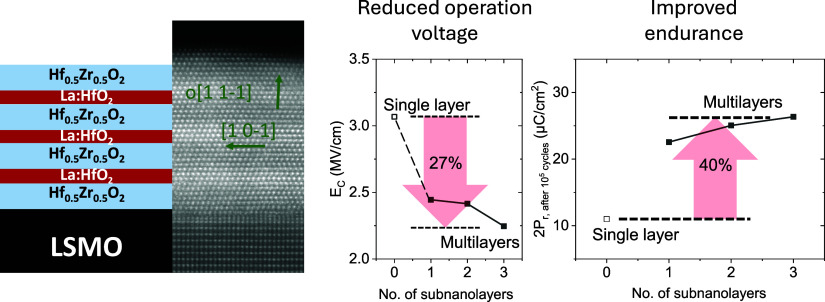

## 1 Introduction

In 2011, ferroelectricity in doped HfO_2_ was reported for the first time.^1^ Ferroelectric HfO_2_ is compatible with complementary metal-oxide semiconductor (CMOS) technology; thus, offering an alternative to conventional ferroelectric materials for nonvolatile memory devices.^2–4^ Relevant properties, such as polarization, permittivity, endurance, retention, and switching time, are determined by the ferroelectric/nonferroelectric phases ratio, thickness, grain size, oxygen vacancies, and interfaces, which are primarily controlled by the dopant atom and concentration, as well as the growth parameters.^2,5^ Therefore, precise control of the microstructure of the film is critical. In recent years, nanolamination has emerged as a promising engineering strategy to enhance ferroelectric performance.^6–19^ Nanolamination consists of the introduction of interlayers of a different materials, usually during atomic layer deposition growth, forming a multilayered system based on ferroelectric doped hafnia. The most studied ones are those obtained combining HfO_2_ and ZrO_2_ layers.^6–8,12–22^ Lehninger et al. showed that the orthorhombic phase ratio, i.e. polarization, was maximized when the interlayer periodicity was reduced to approximately 1 nm.^8^ Reduction of the coercive electric field (E_C_)^23^ and enhancement of the dielectric permittivity (ε_r_)^13–15^ have also been reported in HfO_2_/ZrO_2_ nanolaminates. Regarding the E_C_ reduction, previous works have proposed defects^24^ and the coexistence of multiple phases^12,15^ as possible origins. Similar effects have also been observed in single films of other compositions, and therefore the positive effect of nanolamination alone, regarding E_C_ reduction, cannot be unambiguously established from these previous data.^12,25–30^ In epitaxial films, the use of La as a dopant—accompanied by an increase in the cubic phase fraction—has also been shown to reduce E_C_.^31,32^ Regarding the ε_r_ enhancement, the effects of nanolamination has been attributed to the formation of the tetragonal phase, which occurs at the cost of a reduction in remanent polarization (P_r_). Hf_0.5_Zr_0.5_O_2_ (HZO)/Al_2_O_3_ have also been widely studied. For HZO/Al_2_O_3_ nanolaminates, it is interesting that good functional properties are maintained in films with thicknesses much above 20 nm.^9–11^ However, all these previous works focus on polycrystalline films that usually exhibit a noticeable wake-up effect and fatigue is present. For applications it is mandatory to obtain stable polarization upon electric cycling and thus wake-up and fatigue must be reduced, expanding the plateau region where stable polarization is obtained upon cycling.^33^

In polycrystalline films, sample crystallization is a result of postdeposition annealing, which leads to grainy morphology, significant roughness, and possible chemical interdiffusion. Therefore, the use of layers of different compositions impacts many sample parameters, which makes it very challenging to distinguish if the distinct ferroelectric properties in the nanolaminates are a consequence of modified composition, microstructure, or different electrostatic boundary conditions. The microstructure is much better controlled in epitaxial films fabricated by in situ crystallization processes.^34^ Indeed, in epitaxial HZO/HfO_2_ and HZO/ZrO_2_ bilayers, the columnar growth is not interrupted at the interface, permitting a sharp composition discontinuity without phase change.^35^ Epitaxial multilayers are thus potentially ideal to allow for a better selection of materials and architectures for the enhancement of properties. HfO_2_/HZO epitaxial multilayers showed enhanced dielectric constant and ferroelectric polarization.^36^ However, endurance and retention of these structures were relatively low, inviting the exploration of interlayers with alternative composition. Highly La-doped HfO_2_ is a potential candidate, as the cubic phase is predominant in epitaxial films of this composition and leakage is low.^32^ Motivated by these previous works, we addressed the preparation and characterization of multilayers combining subnanolayers of La(10%):HfO_2_ (LHO) with HZO. Here we report that the multilayers grow with a columnar microstructure across the entire thickness and, interestingly, cubic phase is not present within the detection limit. The samples show high P_r_ and ε_r_, while robust polarization upon cycling is shown related to the reduced E_C_ and leakage current in the multilayers. In addition, good retention, a fast response time of 100 ns, limited by the circuitry of the used setup, and resistive switching as high as 10^8^% are also observed. The results demonstrate that the selection of subnanolayer composition is a key aspect, and that multilayers including highly doped La subnanolayer show remarkable properties.

## 2 Results and Discussion

LHO/HZO multilayers were grown by pulsed laser deposition on SrTiO_3_(001) (STO) substrates buffered with a conducting La_0.67_Sr_0.33_MnO_3_ (LSMO) layer used as an electrode. Three multilayers were fabricated by inserting 1, 2 and 3 0.8 nm thick-LHO into HZO, and these are labeled as M1, M2, and M3, respectively (Figure 1(a,b,c)). A single 9.2 nm HZO film was used as a reference. Thicknesses are extracted from simulation of Laue oscillations present in the X-ray diffraction (XRD) scans shown in Supporting Information Figure S1. Platinum top electrodes are shown as a top planar view in Figure 1(a,b,c), bottom images. XRD θ-2θ scans are shown in Figure 1(d). The peak at 2θ ∼ 30° is at the position of the (111) reflection of the orthorhombic (o) phase while the low intensity peak at 2θ ∼ 34.5° likely corresponds to the (002) reflection of the monoclinic (m) phase, despite the o(002) peak overlapping with this. Note that the peaks ascribed to the o(111) phase are surrounded by additional peaks corresponding to Laue oscillations, indexed in the Figure from −3 to +2. The substrate and LSMO peaks are also observed. As shown in Figure 1(e), the normalized intensity of the peak ascribed to the o(111) phase (I_o(111)_/I_LSMO(002)_) increases with the number of LHO subnanolayers. Interestingly, evidence of more m-phase while introducing the LHO subnanolayers compared with the HZO film is not observed.

**Figure 1:**
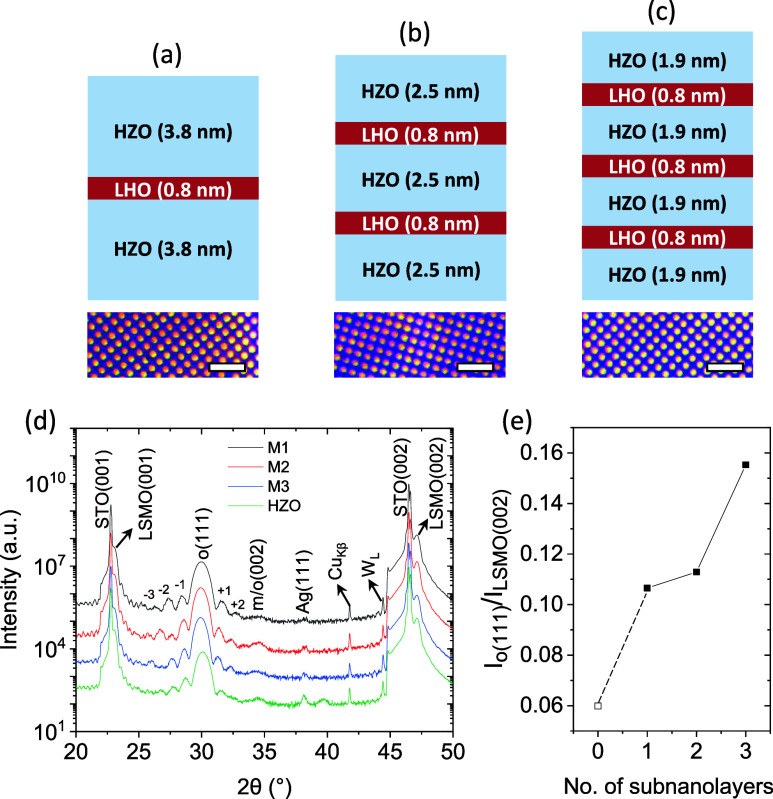
(a, b, c) Schematic representation of the multilayers, featuring 1, 2, and 3 LHO interlayers, respectively. At the bottom are the images of the Pt top electrodes. Scale bars correspond to 100 μm. (d) XRD θ–2θ scans (measured with a point detector) of multilayers and of the single-layer. Numbers correspond to the Laue fringes. (e) I_o(111)_ normalized to I_LSMO(002)_ vs number of subnanolayers.

Figure 2(a) shows low magnification high-angle annular dark field (HAADF) scanning transmission electron microscopy (STEM) images obtained from the M3 sample. The sample thickness is around 10 nm in good agreement with results from Laue simulations. The contrast between LSMO and the multilayer indicates a sharp interface. Additionally, a clear contrast among each layer of the multilayer is clearly visible. Figure 2(b) shows a high magnification image of the region enclosed in red in Figure 2(a), overlaid with a compositional STEM energy dispersive X-ray spectroscopy (EDS) map of the structure, where the alternate contrast of Hf in red and Zr in blue is evident. The multilayer nature of the M3 sample is confirmed by the vertical compositional profile shown in Figure 2(c), which evidence Hf/Zr alternate contrast between the HZO and LHO layers. Note that the gradient of composition across the film is limited by the experimental resolution and does not reflect the actual sharpness of the interfaces. Figure 2(d) further evidences the presence of o(111) regions (green and red shadings) in addition to minority m(002) regions (blue shading). Grains corresponding to cubic phase have not been detected. Figure 2(e,f) show atomic-resolution images of selected regions of Figure 2(d), where different grains with m⟨002⟩ and o⟨111⟩ out-of plane orientations have been indexed. In these images, it is confirmed that identified phases show columnar microstructure across the film thickness. Therefore, the LHO subnanometric layer exhibits the same phase as the grain on which it crystallizes, in contrast to the cubic phase formation in homogeneous films of pure LHO.^32^ This is because in multilayers, the formation of an interface with a phase discontinuity (cubic on orthorhombic, for example) would be costly. Despite the lower energy of the cubic phase in LHO, the volume fraction of subnanometric LHO interlayers is low (10.9, 17.6, and 24.0% in M1, M2, and M3, respectively), and the interfacial energy contribution predominates over the volume energy contribution. It is worth noting that the boundaries—whether between orthorhombic columns or between orthorhombic and monoclinic columns—are highly coherent and that defects are not observed in contrast with single layer HZO, where grain boundaries are blurrier and defects, despite in low amount, are present.^37^

**Figure 2:**
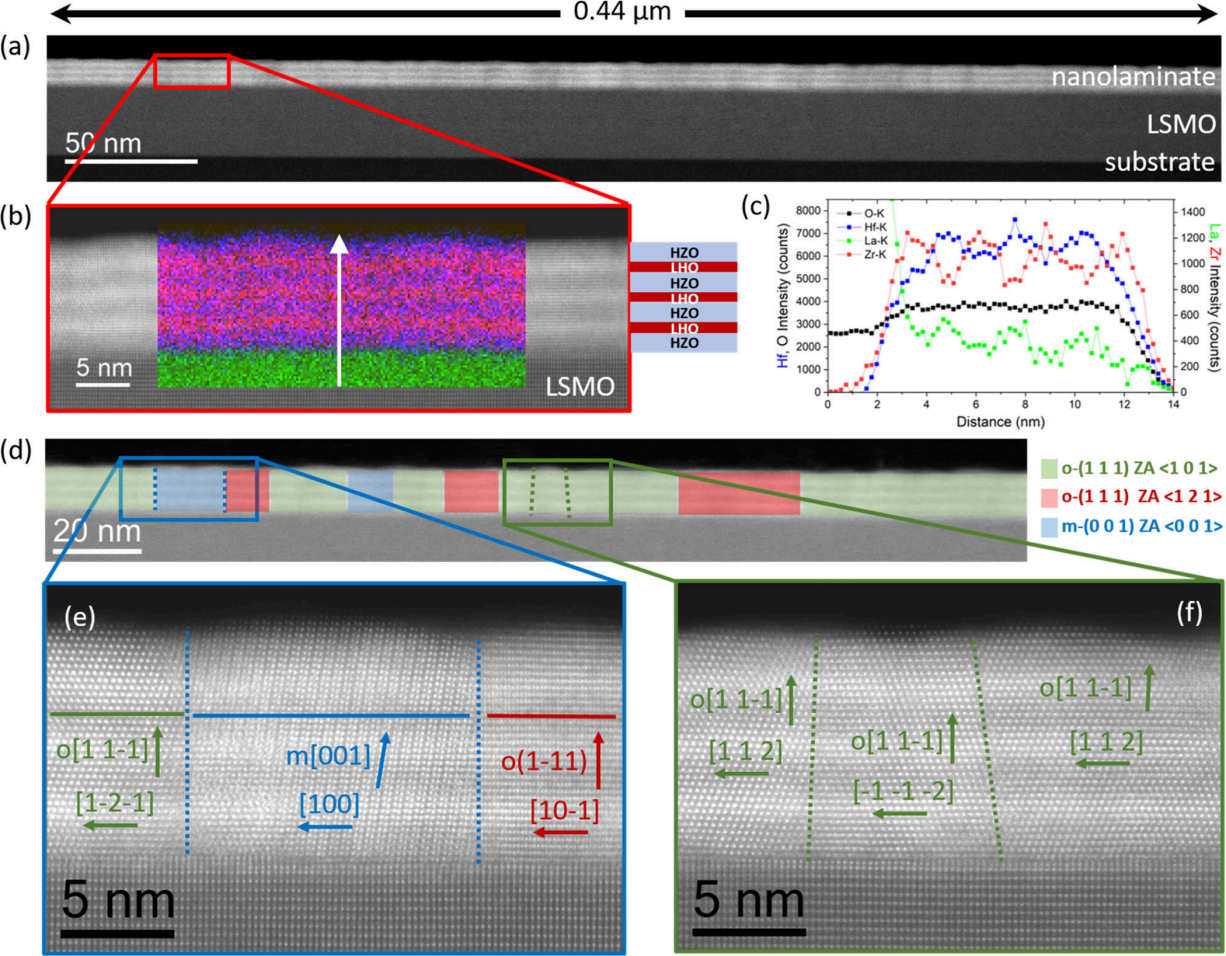
(a) Low magnification cross sectional HAADF-STEM image obtained from the M3 sample. (b) Close-up image of the region marked in red in (a) with an overlay of the STEM-EDS compositional map collected in that area, where Hf is depicted in red, Zr in blue and La in green. A sketch of the HZO/LHO multilayer is also included. (c) Line profile of the chemical map, collected along the white arrow marked in (b), integrating ∼10 nm along the horizontal direction. (d) High magnification image of the same specimen, in which regions identified as orthorhombic and monoclinic phases are marked according to their crystal phase (o or m) and zone axis (ZA) orientation. (e, f) Atomic resolution HAADF images of the representative regions, enclosed in blue and green rectangles, respectively, in (d).

Polarization versus electric field (P-E) and current versus electric field (I-E) loops obtained using dynamic leakage current compensation (DLCC) method are shown in Figure 3(a) and 3(b), respectively. At an applied electric field close to 5.8 MV/cm, at which clear saturation is observed, all the films exhibit similar P_r_ values in the range of 17–23 μC/cm^2^. Positive-up-negative-down (PUND) measurements reveal similar polarization values (Figure S2 in Supporting Information). Therefore, the comparison of the P_r_ values among the different samples summarized in Figure 3(c) indicates that P_r_ does not show any significant dependence on the number of subnanolayers. Note that this agrees with the similar piezoelectric amplitude response of the three samples as shown in Supporting Information Figure S3. Notably, E_C_ in multilayers is reduced by around 25% in comparison to that of the single-layer HZO, reaching 27% for M3 sample (Figure 3(d)). This will directly impact on the larger amount of switched polarization and endurance, as far as, lower E_C_ means that saturation can be reached with a smaller electric field compared to a sample with a higher E_C_, keeping the applied field far from the breakdown limit.

**Figure 3:**
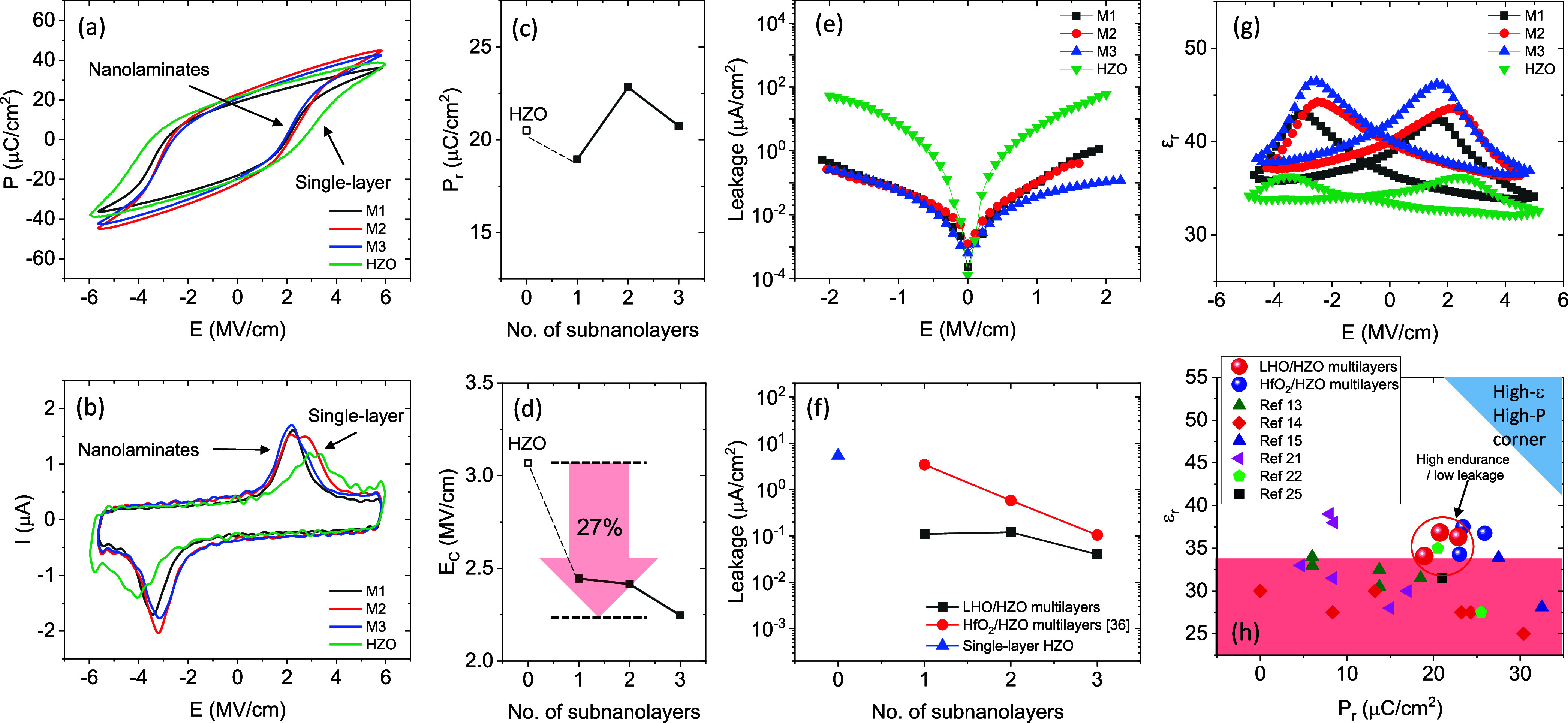
(a, b) P-E and I-E obtained by DLCC, respectively. (c, d) Dependence of P_r_ and E_C_ on the number of subnanolayers, respectively. (e) Comparison of leakage in multilayers and single-layer HZO. (f) Leakage current at 1 V as a function of the number of LHO subnanolayers (black squares) and single-layer HZO (blue triangle). Reported data for epitaxial HfO_2_/HZO multilayers (blue circles)^36^ are included. (g) ε_r_-E loops and (h) ε_r_ at saturation vs P_r_. Reported data of epitaxial HfO_2_/HZO multilayers,^36^ and polycrystalline multilayers are included.

The insertion of LHO subnanolayers results also in reduced leakage (0.11, 0.12, 0.04 μA/cm^2^ at 1 MV/cm for M1, M2, M3, respectively) compared to the single-layer HZO (5.4 μA/cm^2^, Figure 3(e)). We ascribe this result to the good microstructure of the multilayers revealed by the STEM characterization, but it can be also probably related to the selected composition for interlayers, since highly La doped single films show reduced leakage.^32^ This is further confirmed by the fact that LHO/HZO shows reduced leakage compared with HfO_2_/HZO multilayers (as summarized in Figure 3(f)),^36^ therefore by the introduction of La as a dopant at the HfO_2_ subnanolayers improved leakage is achieved. Although no intermixing was observed in the STEM analysis, interdiffusion between interlayers not detectable due to the limited experimental sensitivity could, in principle, also allow the formation of La-doped HZO, which has been reported to exhibit reduced leakage^29^ owing to charge compensation. This mechanism could therefore also contribute to the observed leakage reduction. The ε_r_-E loops are shown in Figure 3(g), with the ε_r_ values increasing for M1, M2, and M3. The butterfly shape indicates the ferroelectric character of the samples. Figure 3(h) presents the relationship between ε_r_ (measured at saturation) and P_r_, with data from literature of polycrystalline nanolaminates^13–15,21,22^ and epitaxial multilayers^35,36^ also shown. ε_r_ at saturation is 34, 36, and 37 for M1, M2, and M3, respectively. LHO/HZO epitaxial samples display ε_r_ values that are significantly higher compared to ε_r_ values of polycrystalline nanolaminates^13–15,21,22^ and comparable to those of previous epitaxial HfO_2_/HZO heterostructures,^36^ but performing lower leakage. In this latter study,^36^ it was discussed that presence of o(001) grains, addionally to the o(111) grains, can result in the observed permittivity improvement, an scenario that could also apply here, although STEM characterization is not conclusive due to the lack of statistical significance.

P-E loops upon cycling for the representative M3 sample are show in Figure 4(a), where it can be observed that loops are virtually indistinguishable up to 10^5^ cycles. The endurance characteristics of all the multilayers, measured while cycling at 4.8 MV/cm at 100 kHz, is shown in Figure 4(b). Note that an electric field smaller than the saturation field has been used to avoid fast breakdown. Raw data for all samples is shown in Supporting Information Figure S4. The multilayers exhibit no wake-up and nearly no fatigue up to around 10^5^ cycles, being M3 the sample that shows less reduction of P_r_ after a larger number of cycles. The absence of wake-up is a common characteristic of epitaxial films crystallized at high temperature,^34^ which is ascribed to the reduced number of defects and the absence of significant amount of tetragonal phase in these films. Note that empty symbols of Figure 4(b) account for 2P_r_ values extracted from loops where no ferroelectric current switching peaks are observed. The better endurance of multilayers compared with the single film can be also clearly inferred by the direct comparison of P_r_ values collected after 10^5^ cycles shown in Figure 4(c). Polarization window is enhanced by approximately 40%, if evaluated after 10^5^ cycles compared to that of the single layer, owing to the reduced E_C_ of multilayers. As aforementioned, E_C_ reduction results in larger P_r_ if under saturation electric field is used, finally leading to better endurance as observed. The gradual decrease of the dielectric permittivity upon cycling (Supporting Information Figure S5) suggests that the fatigue can be linked to transformation to monoclinic phase with lower permittivity.^7,38^ The origin of the E_C_ reduction can be diverse, as proposed in the literature: a lower defect concentration^24–28,39^ and the presence of nonferroelectric phases^12,15,29,30^ can facilitate switching. Strain effects can also play a relevant role. In our case, the multilayers, compared to the single layer, show (i) lower leakage current, which is an indication of a reduced defect density, (ii) contributions from (001)-oriented orthorhombic grains or cubic grains coexisting with (111)-oriented orthorhombic ones, as revealed by structural and dielectric permittivity characterization, and (iii) the strain state differs, as evidenced by the d_o(111)_ values of 2.97, 2.98, 2.99, and 2.99 Å for the single layer, M1, M2, and M3 samples, respectively (Supporting Information Figure S1). Therefore, it is difficult to unambiguously identify the origin of the E_C_ reduction in our system, and it is possible that all three mechanisms cooperatively contribute.

**Figure 4:**
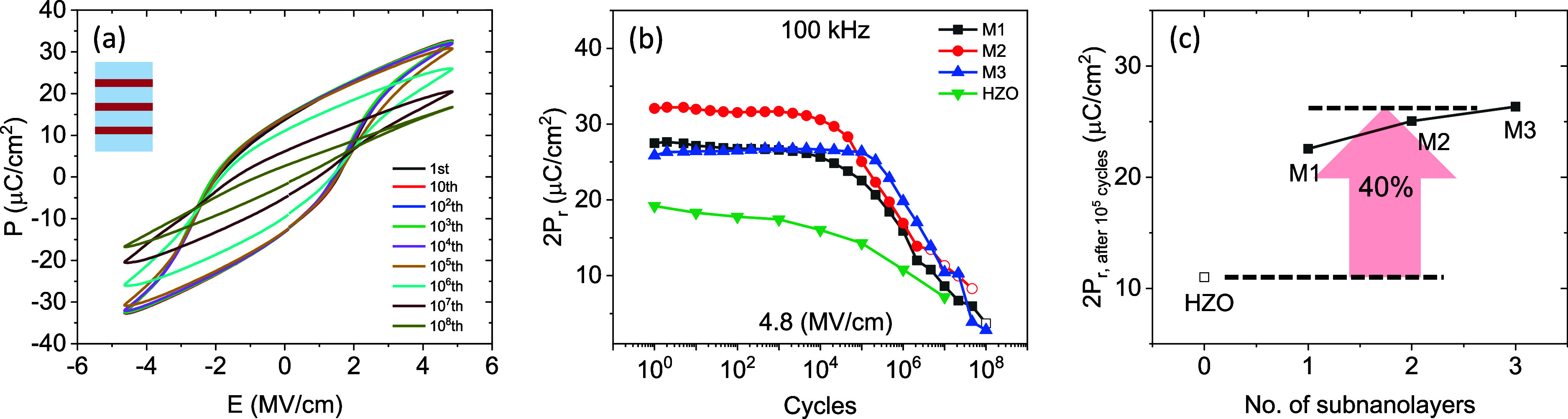
(a) P-E loops of M3 multilayer obtained from endurance measurements at 4.8 MV/cm after the indicated number of cycles. (b) 2P_r_ versus number of cycles for the multilayers and HZO film. (c) 2P_r_ versus number of subnanolayers evaluated after 10^5^ cycles.

The retention characteristics of the multilayers and HZO films were examined under an electric field (5.4 MV/cm) slightly below saturation (Figure 5(a)). It is evident that the multilayers exhibit significantly higher retention. The experimental data were fitted using a rational decay model, expressed as *P*_*r*_ = *P*_0_ *t*_*d*_^‑*n*^.^40^ Supporting Information Figure S6 shows experimental data fitted to the logarithmic dependence model, following the equation $P_{r}=P_{0}-n\log\left(\frac{t_{d}}{t_{0}}\right)$ .^41,42^ The multilayers exhibit significantly better retention performance, with extrapolated P_r_ values ranging from 13 to 15 μC/cm^2^ and polarization retention between 78% and 89% as is shown in Figure 5(b), in which P_r_ extrapolated values using both models are plot. For representative M3 sample, retention data has been collected up to ≈ 4 × 10^6^ seconds (≈ 1 month). It is observed that these data follow the extrapolated trend. In contrast, the HZO film shows inferior retention, with a P_r_ of approximately 6.4 μC/cm^2^ and a P_r_/P_0_ of around 55% after 10 years. Supporting Information Figure S7, shows n dependence on sample for both models, where lower n values for multilayers indicate better retention. Equivalent characterization performed at 85 °C is shown in Supporting Information Figure S8, where it can be observed that irrespective of the used fitting model, extrapolated P_r_ is above 7.5 μC/cm^2^ for all multilayers, but much smaller for single-film.

**Figure 5:**
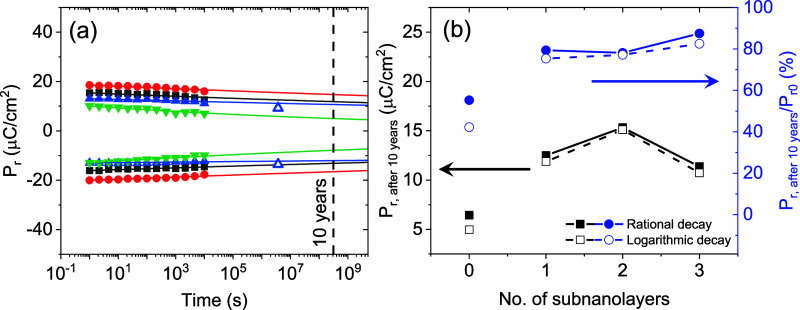
(a) Retention plot after poling at 5.4 MV/cm. Lines through data correspond to fitting using rational equation. The blue empty triangles correspond to P_r_ evaluated after 1 month for M3 sample. (b) P_r_ and P_r_/P_0_ (%) extrapolated to 10 years as a function of number of interlayers using rational and logarithmic equations.

Switching spectroscopy characterization was carried out to better understand the performance of the multilayers. The applied pulse train is shown in Supporting Information Figure S9. Loops obtained after prepoling voltage of indicated duration (positive and negative) are shown in Figure 6(a–c) for M1, M2, and M3, respectively. In Figure 6(d–i), switched polarization (ΔP) versus τ_w_ is plotted for all the samples and for both polarities. First, it is observed that faster switching always occurs for positive polarity because of the presence of the negative imprint electric field (E_imp_ ≈ −0.5 MV/cm) as inferred from the shift of the loop toward negative electric field values in the P-E loops of Figure 3(a). It can be observed that polarization switching (ΔP = 12 μC/cm^2^ for M2 for positive polarity) occurs at times as fast as 100 ns, limited by the circuit time constant.^36^ Larger ΔP is reached after around 500 ns, faster than in equivalent epitaxial films of similar thickness.^36^ Second, it is observed that similar ΔP values are obtained for the explored τ_w_ comparing data collected using the same E for the three samples. However, a slightly higher ΔP for M2 is observed for τ_w_ < 1000 ns and E > 5 MV/cm.

**Figure 6:**
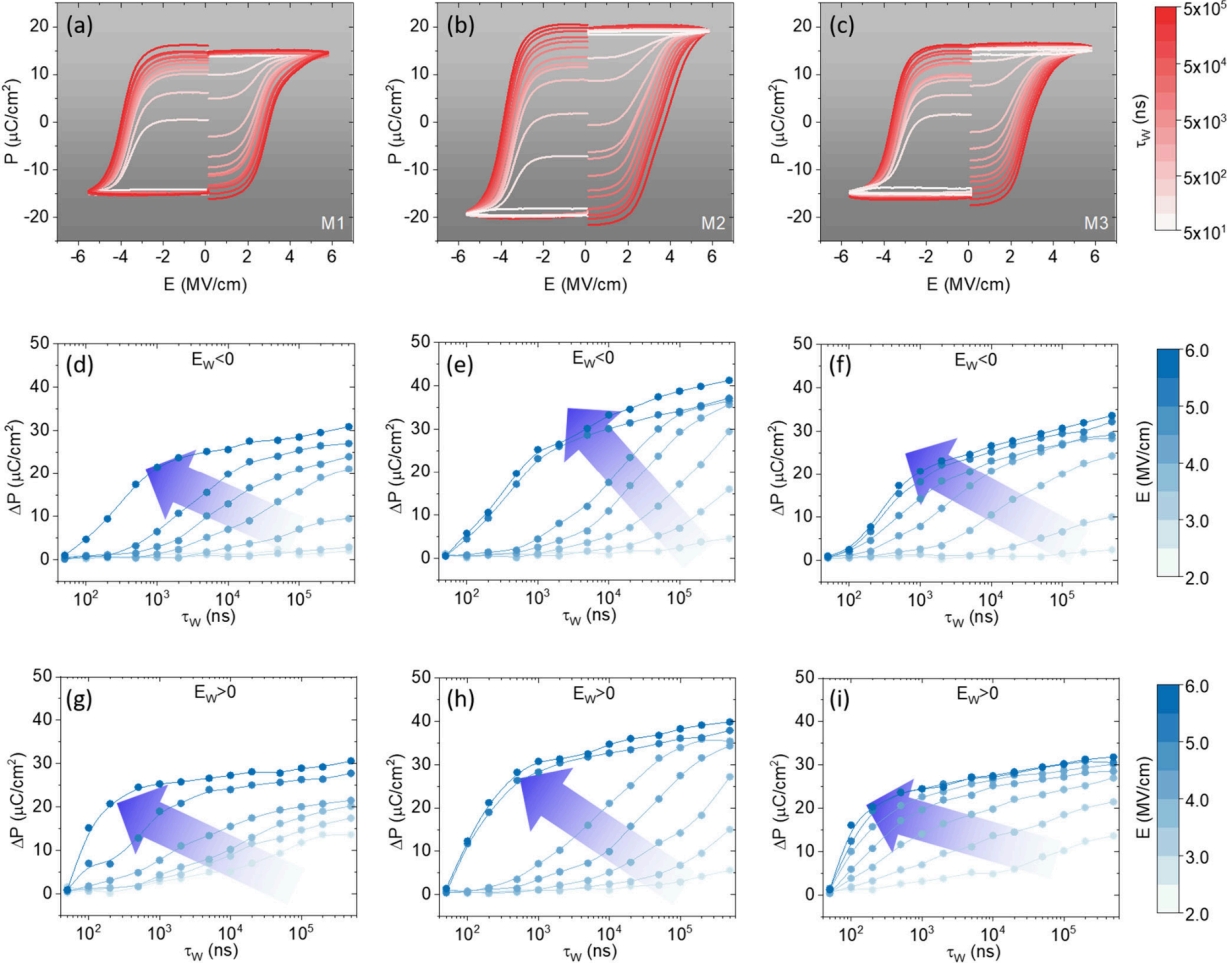
(a–c) P-E loops obtained from switching spectroscopy measurements for multilayers M1, M2, and M3, respectively, at 5.8 MV/cm, with writing pulse widths from 50 ns to 500 μs, at 5.4 MV/cm. (d–f) ΔP as a function of the writing pulse width at various negative writing pulse amplitudes, from −5.8 to −2.5 MV/cm, for M1, M2, and M3, respectively. (g–i) Corresponding curves of (d–f) under positive writing pulse conditions from 2.5 to 5.8 MV/cm.

Figure 7(a) shows current density vs electric field curves collected up to approximately 6.5 MV/cm. In the negative electric field branch, a conductivity increase is observed near E ≈ −6 MV/cm, resembling the conductance increase occurring during the filament formation process in HfO_2_ films.^43–50^ Note that the samples become leaky after the soft-breakdown process, and reliable P–E loops can no longer be obtained (see Supporting Information Figure S10). Figure 7(b) shows electroresistance (ER), calculated as $\frac{R_{HIGH}-R_{LOW}}{R_{LOW}},$ as a function of electric field. It can be observed that the ER is significantly larger for negative electric field ER is the highest for the M2 film, exceeding 10^8^%, compared to the other multilayers (10^6^%). Note that the ER heavily decays with cycling (Supporting Information Figure S11). This indicates that its appearance is not related to ferroelectric switching, whose robustness upon cycling is much larger (Figure 4). Figure 7(c,d) show current density versus the square root of the applied electric field at the negative electric field region in the low and high resistance states, respectively. The linear dependence (indicated by dashed lines) observed in Figure 7(c) for the low resistance state agrees with the thermionic current conduction mechanism.^51^ In contrast, Figure 7(d) shows that thermionic current is not present for the high resistance state, indicating that current is limited by the bulk of the multilayer rather than its interfaces. In brief, remarkable differences in conduction mechanisms in multilayers are not observed compared to those in the single layer, indicating that the bulk-like response of the multilayer dominates the soft-breakdown mechanism and that the reduced leakage of the multilayers does not have a significant impact on it.

**Figure 7:**
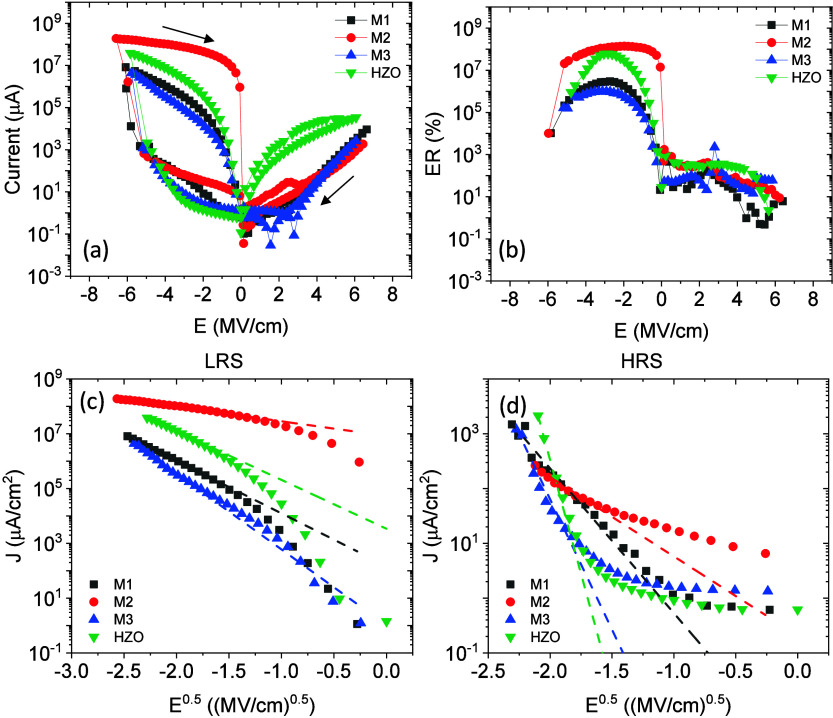
(a) Current versus electric field of the multilayers compared to the single layer HZO. (b) ER (%) extracted from (a). (c) and (d) correspond to J-E curves of the LRS and HRS in the negative electric field region of (a), respectively.

## 3 Conclusions

Epitaxial multilayer heterostructures show columnar microstructure and polarization is comparable to single films. The cubic phase is not detected in multilayers, contrary to 10% La doped HfO_2_ films, where the cubic phase is predominant. The insertion of LHO subnanolayers results in E_C_ and leakage current reduction, leading to improved endurance of LHO/HZO multilayers compared to single HZO films. Permittivity also increases in multilayers, and fast switching time and good retention are measured. The dominant conduction mechanism is thermionic emission for the high resistance state, which can be switched to a lower resistance state at 6 MV/cm showing ER as high as 10^8^ %. In brief, we demonstrate that the introduction of LHO subnanolayers in multilayered structures has a direct positive effect on functional properties.

## 4 Experimental Section

LHO/HZO multilayers were grown by pulsed laser deposition on STO substrates buffered with a conducting LSMO layer used as an electrode. LSMO electrodes, with a thickness of 25 nm, were deposited under dynamic oxygen pressure (PO_2_) of 0.1 mbar and at a substrate temperature (T_s_) of 700 °C. Subsequently, LHO and HZO were deposited under the same PO_2_ of 0.1 mbar but at an elevated T_s_ of 800 °C. LHO subnanolayers, 0.8 nm thick, were inserted into HZO. Three multilayers were fabricated with 1, 2, and 3 LHO subnanolayers, labeled as M1, M2, and M3, respectively (Figure 1(a,b,c)). A single HZO film was also grown. The total thicknesses of the M1, M2, and M3 multilayers and the single film were 8.3, 9.5, 9.8, and 9.2 nm, respectively, as determined by Laue fringes simulation shown in Supporting Information Figure S1. From the total thicknesses measured, the thickness of each individual layer was calculated by assuming a constant growth rate, and these are shown in Figure 1(a,b,c). Platinum top electrodes, 20 nm thick and 20 μm in diameter, were deposited onto the samples using DC magnetron sputtering through a stencil mask, and these are shown as a top planar view in Figure 1(a,b,c), bottom images.

The crystal structure was characterized using XRD with Cu_Kα_ radiation using a Bruker D8 Discover diffractometer equipped with a point detector. Atomic-scale structural analysis of selected films was performed by (STEM in HAADF imaging mode. A Thermo Fisher Titan 60–300 microscope equipped with a high brightness Schottky field emission gun and a CETCOR probe-corrector (CEOS Gmbh) was operated at 300 kV to provide a probe size below 0.1 nm. EDS was performed in an Ultim Max TLE10 system by Oxford Instruments. Cross sectional lamellas of the specimens, cut along (110) planes of the STO substrate were prepared by focused ion beam milling in a Thermo Fisher Helios 650 Nanolab. STEM image simulations were carried out with the Dr. Probe software package.^52^

Ferroelectric polarization loops, retention, and endurance were measured at room temperature using an AixACCT TFAnalyser3000 platform in a top-bottom configuration,^53^ with the bottom LSMO electrode grounded and the top Pt electrode biased. To compensate for leakage in the measured polarization loops, the LCC and PUND methods were employed.^54,55^ Retention measurements were conducted using a poling electric field of 5.4 MV/cm, i.e. slightly lower than the saturation electric field, with a rise/fall time and pulse width of 0.25 ms, followed by reading the polarization state with the opposite state protocol and PUND sequence. Endurance tests were carried out at 4.8 MV/cm using 100 kHz bipolar electric field pulses, with remanent polarization determined by averaging positive and negative polarization values from DLCC loops measured at 1 kHz under the same electric fields.

The specific pulse train used for switching dynamics experiments is shown in Supporting Information Figure S9. First, a preswitching pulse is applied. This is long enough to ensure polarization saturation at 5.4 MV/cm. Afterward, a rectangular switching pulse of plateau time (τ_w_) is applied. The rectangular switching pulse has rise and fall times of 25 ns. After 1 s, a pulse of opposite to the writing polarity is used for polarization reading. The switched polarization (ΔP) is calculated from the subtraction of the values measured during X and U (i.e., X-U) or X and D (X-D),^56^ where X corresponds to the reading pulse. The leakage current was measured using a 2 s integration time, with data averaged during both increasing and decreasing voltage sweeps. Capacitance (C) loops were measured using an excitation voltage of 0.3 V at a frequency of 50 kHz. The dielectric permittivity (ε_r_) loops were then calculated from the capacitance values using the C = ε_0_ε_r_A/t relation, where A corresponds to the electrode area and t to the film thickness.

## Supplementary Material



## Data Availability

The data that support the findings of this study are available from the corresponding author upon reasonable request.

## Notes

Additional XRD characterization, PUND P–E loops, piezoelectric response, endurance loops, permittivity, additional experimental details, and leakage current additional data (PDF)
