# Supplementary material for: Interface Engineering Using Multiple La-Doped HfO2 Epitaxial Subnanolayers To Improve the Ferroelectric Properties of Hf0.5Zr0.5O2 Films
Source: ACS Appl Electron Mater. 2025 Dec 26;8(1):327–36. doi: 10.1021/acsaelm.5c02016 (PMC12805815; doi:10.1021/acsaelm.5c02016)
Supplement: Supplementary file 1 [file el5c02016_si_001.pdf]

Supporting Information

Interface engineering using multiple La-doped HfO_2 epitaxial subnanolayers to improve the ferroelectric properties of $Hf_{0.5}Zr_{0.5}O_2$ films

Mehrdad Ghiasabadi Farahani,[1] Tingfeng Song,[2,3] César Magén[4,5], Jingye Zou,[1] Florencio Sánchez,[1,*] Ignasi Fina[1,*]

[1]*Institut de Ciència de Materials de Barcelona (ICMAB-CSIC), Campus UAB, Bellaterra 08193, Spain*

[2]*Department of Applied Physics, The Hong Kong Polytechnic University, Hong Kong, China*

[3]*Joint Research Center of Microelectronics, The Hong Kong Polytechnic University, Hong Kong, China*

[4]*Instituto de Nanociencia y Materiales de Aragón (INMA), CSIC-Universidad de Zaragoza, 50009 Zaragoza, Spain*

[5]*Departamento de Física de la Materia Condensada, Universidad de Zaragoza, 50018 Zaragoza, Spain*

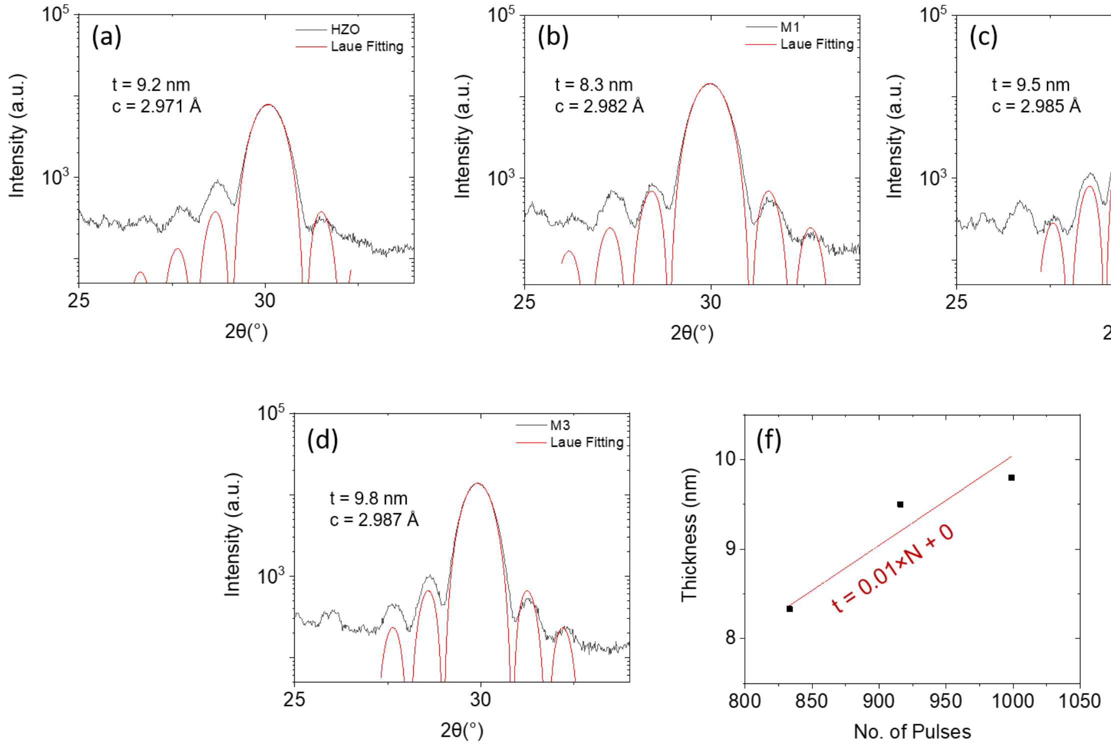

Figure S1. *(a-d) XRD θ–2θ scans of the HZO, M1, M2, and M3. Red lines indicate Laue fringes simulation. (f) Thicknesses of the samples calculated using the Laue fringes simulation of panels (b-d) vs. the number of laser pulses in the pulsed laser deposition process. Growth rate has been assumed to be the same for LHO and HZO and thus the thickness of each layer has been calculated.*

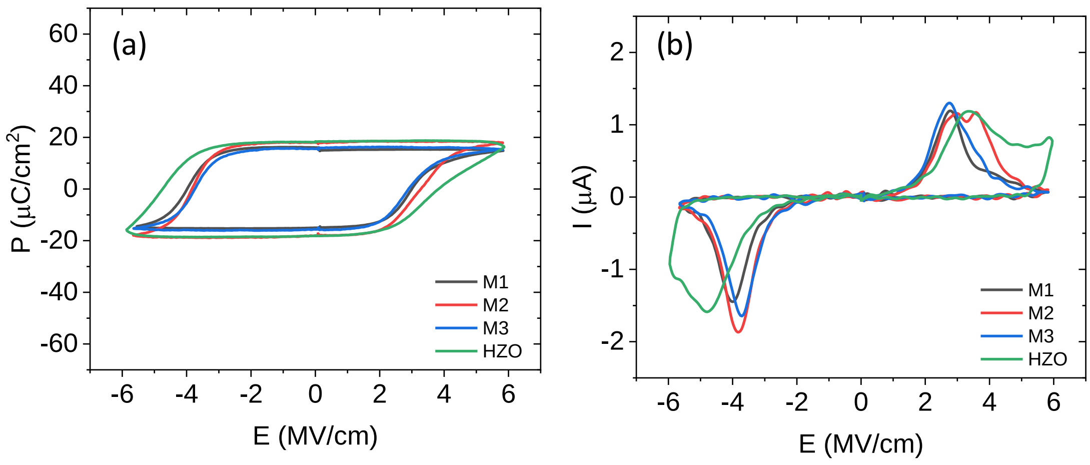

***Figure S2**. (a,b) P-E and I-E obtained by PUND, respectively.*

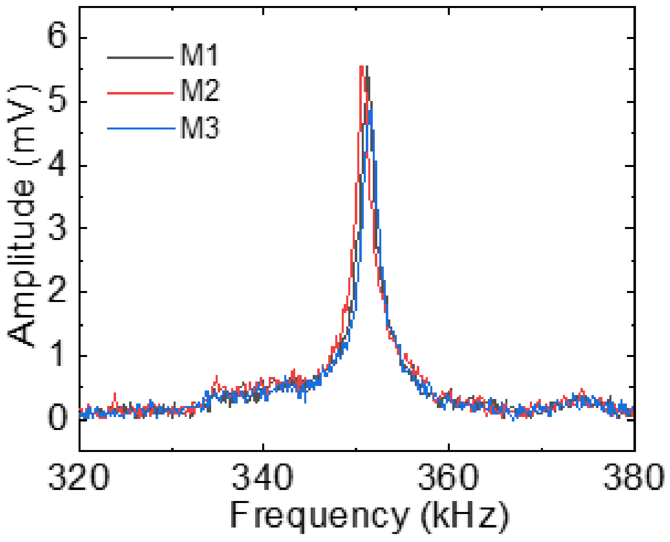

***Figure S3.** Resonance spectra collected at the surface in the as-grown region for M1, M2, and M3. It can be observed a similar piezoelectric response for all the films in agreement with their similar P_r.*

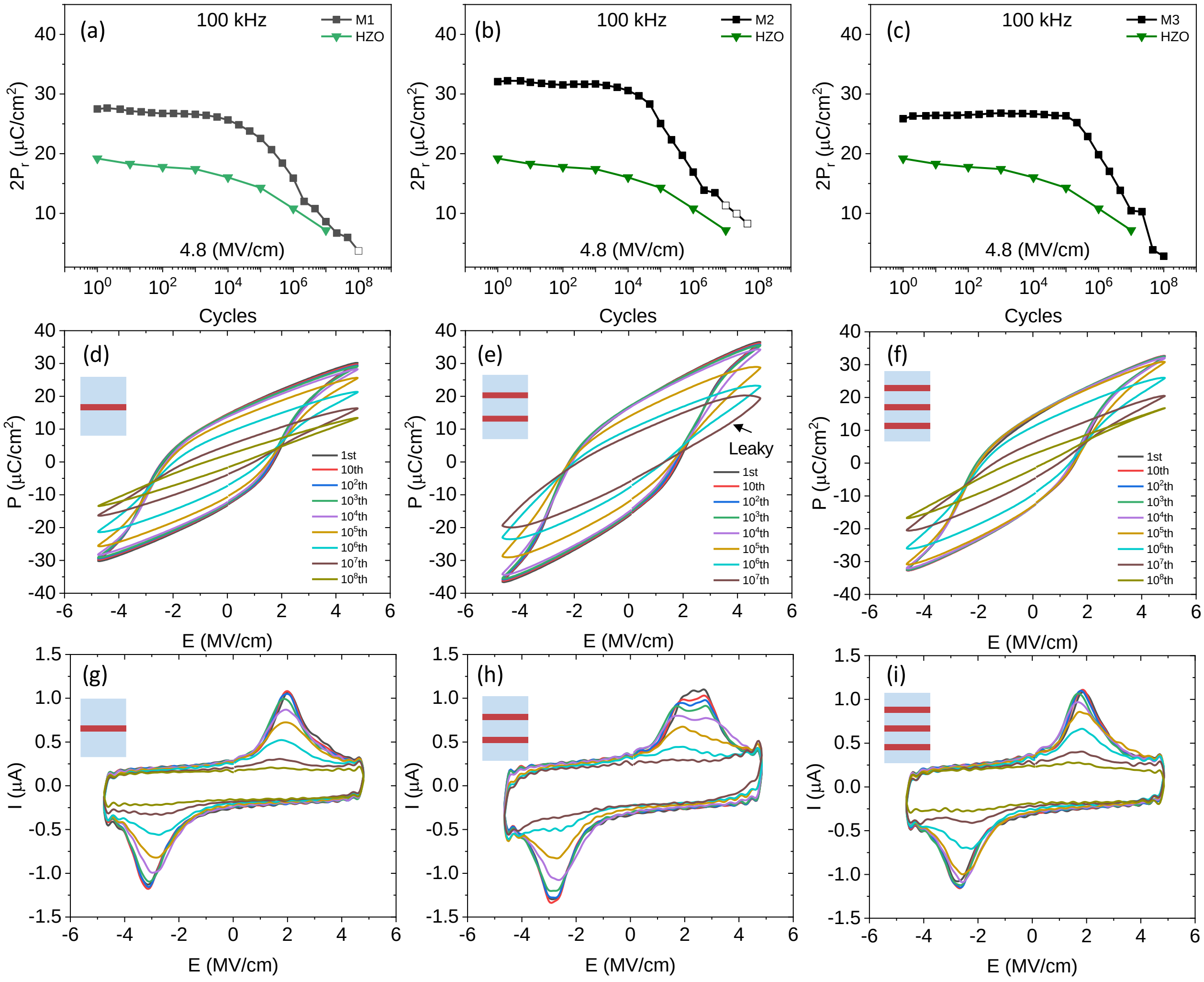

Figure S4. *(a-c) Endurance measurements for M1, M2, and M3 samples, respectively. Empty symbols indicate no ferroelectric switching current peak in the I-E curve of panels (g-i) (d-f) P-E loops of M1, M2, and M3, respectively, after the indicated number of cycles at 4.8 MV/cm. (g-i) I-E curves of the loops shown in (d-f), respectively. All films show clear current ferroelectric switching peaks except for the M2 sample that displays round-shaped loops (Figure S4(e)) and an absence of any ferroelectric switching peaks (Figure S4(h)) after 10^6 cycles. Note that empty symbols of Figure 4(c) account for $2P_r$ values extracted from loops where no ferroelectric current switching peaks are observed, and therefore are mainly contributed by extrinsic effects.*

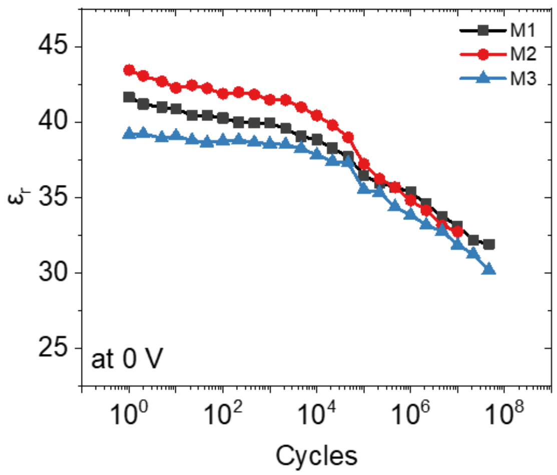

Figure S5. *ε_r at 0 V as a function of the number of. Note the small variation of ε_r compared to data show in **Figure 3** due to the absence of sample prepoling during the cycling and the concomitant small differences in the sample polarization state.*

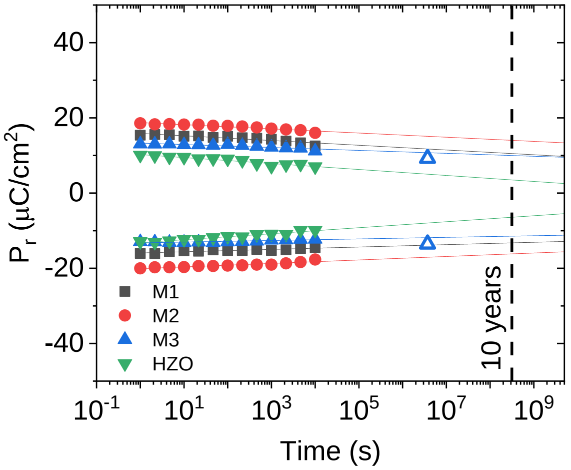

Figure S6. *Retention plot after poling at 5.4 MV/cm. Lines through data correspond to fitting using logarithmic equation $P_r = P_0 - n.log(\frac{t_d}{t_0})$. The blue empty triangles correspond to P_r evaluated after 1 month for M3 sample. Extrapolated P_r and P_r/P_0 (%) values are shown in Figure 5(b).*

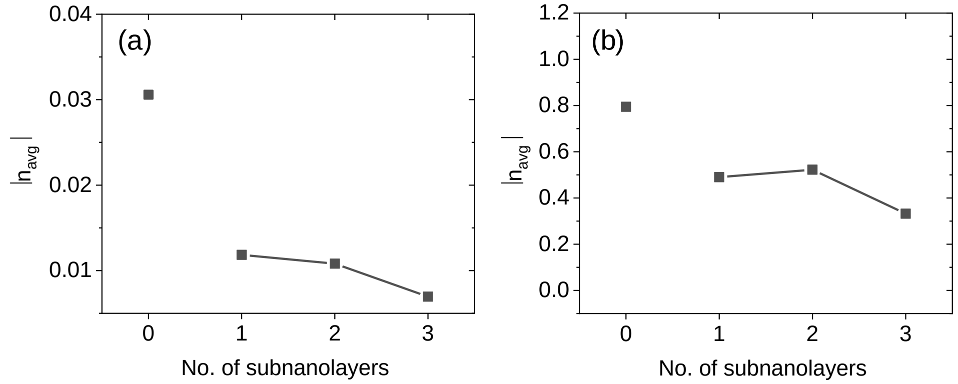

Figure S7. *(a,b) Retention n parameters as a function of the sample for rational and logarithmic models, respectively.*

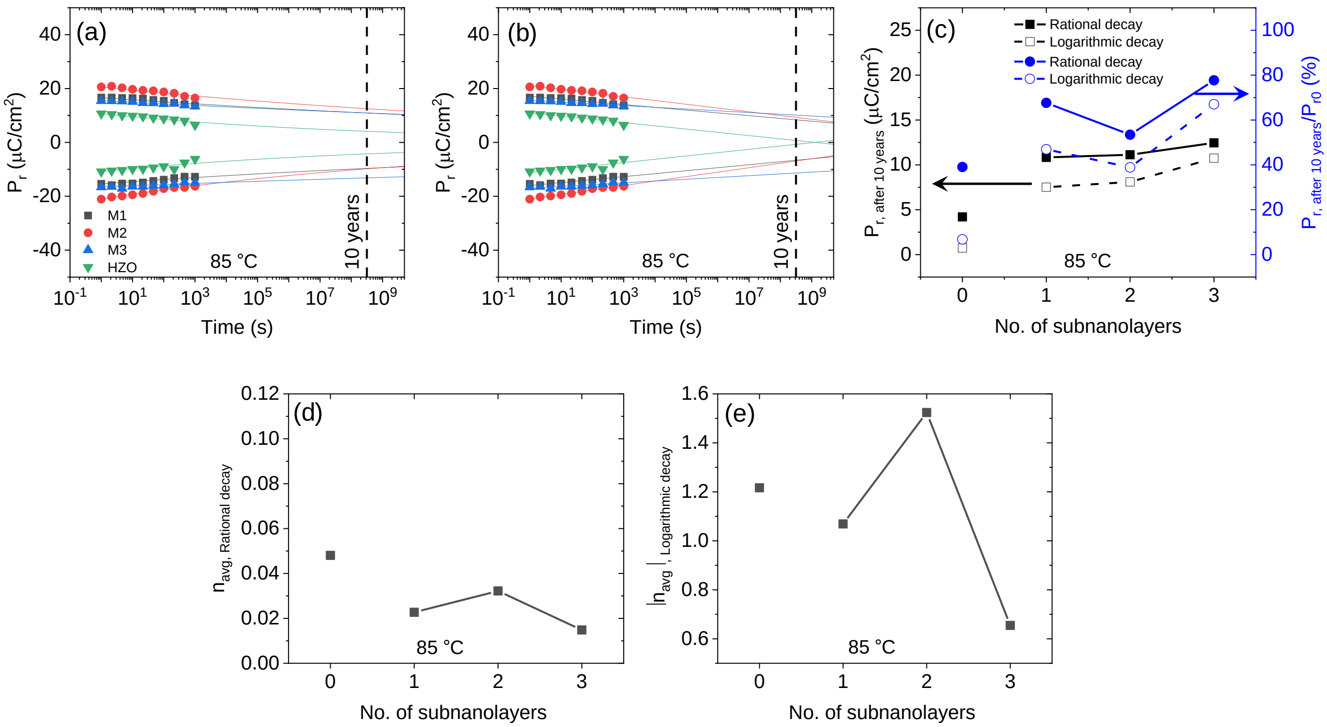

Figure S8. *(a,b) Retention at 85°C plot after poling at 5.4 MV/cm with rational and logarithmic fittings, respectively. (c) P_r and P_r/P_0 (%) extrapolated to 10 years for both models. (d,e) n values at 85°C for rational and logarithmic models, respectively.*

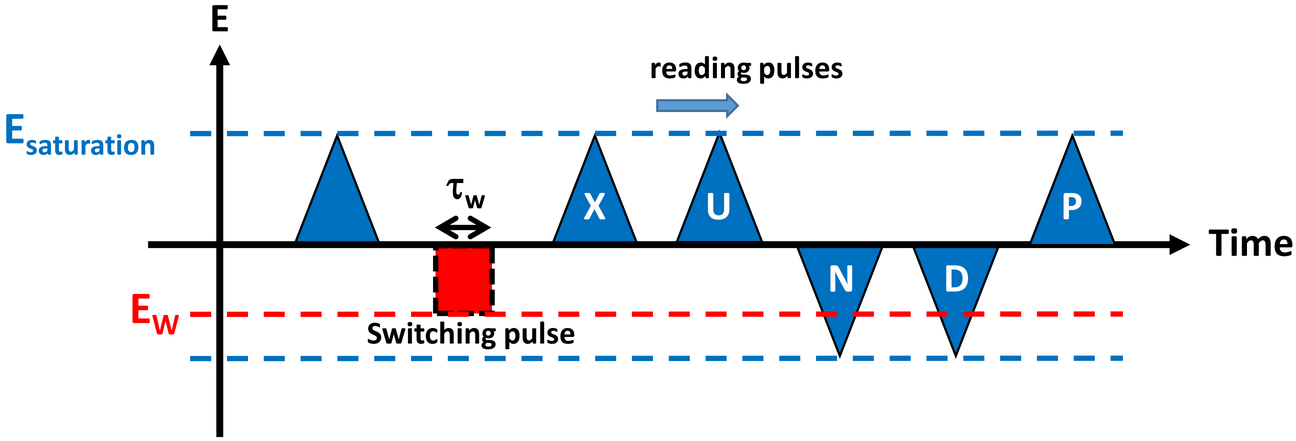

Figure S9. *Schematic illustration of the pulse train employed to characterize the switching dynamics.*

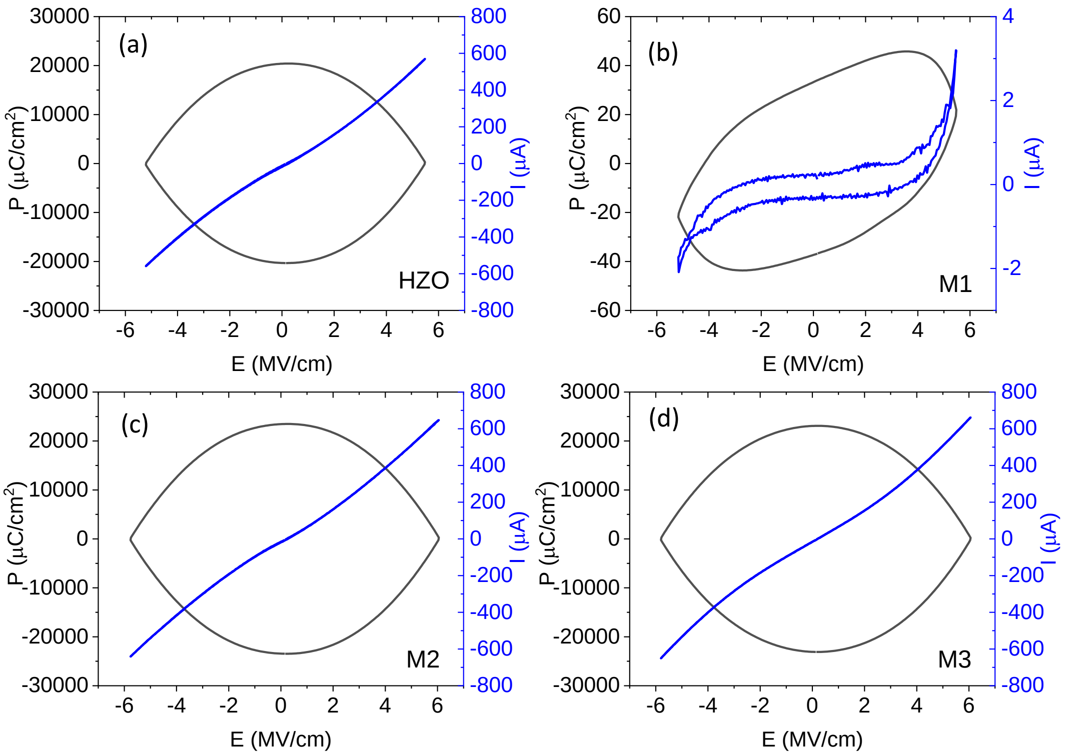

Figure S10. *(a,b,c,d) P-E and I-E loops after soft break down, measurement included in Figure 7 of the main text, for single-layer HZO, M1, M2 and M3, respectively.*

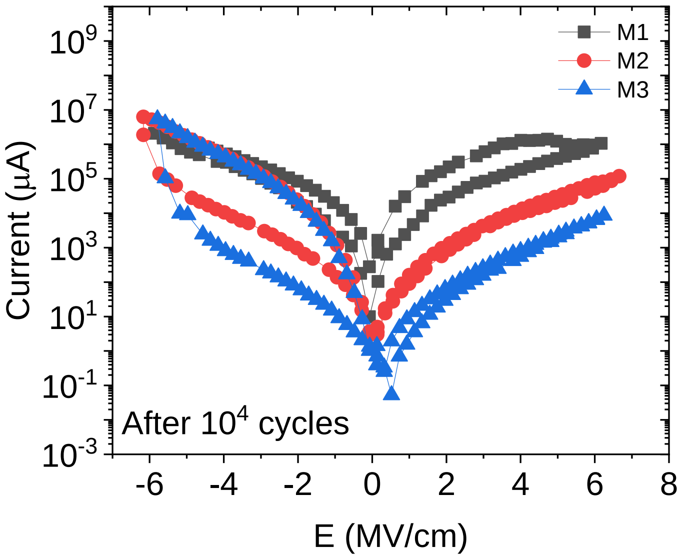

***Figure S11**. Leakage curves of the M1, M2, and M3 samples after 10^4 cycles.*
